# HIV Capsid Protein Genetic Diversity Across HIV-1 Variants and Impact on New Capsid-Inhibitor Lenacapavir

**DOI:** 10.3389/fmicb.2022.854974

**Published:** 2022-04-12

**Authors:** Paloma Troyano-Hernáez, Roberto Reinosa, África Holguín

**Affiliations:** HIV-1 Molecular Epidemiology Laboratory, Department of Microbiology, Instituto Ramón y Cajal de Investigación Sanitaria (IRYCIS), Hospital Universitario Ramón y Cajal, CIBER en Epidemiología y Salud Pública (CIBERESP), Red en Investigación Translacional en Infecciones Pediátricas (RITIP), Madrid, Spain

**Keywords:** HIV-1, p24, variants, conservation, lenacapavir, capsid

## Abstract

The HIV p24 capsid protein has an essential, structural, and functional role in the viral replication cycle, being an interesting target for vaccine design, diagnostic tests, and new antiretroviral drugs (ARVs). The HIV-1 variability poses a challenge for the accuracy and efficiency of diagnostic and treatment tools. This study analyzes p24 diversity among HIV-1 variants and within its secondary structure in HIV-1 M, O, P, and N groups. All available HIV-1 p24 nucleotide sequences were downloaded from the Los Alamos HIV Sequence Database, selecting 23,671 sequences belonging to groups O, N, P, and M (9 subtypes, 7 sub-sub types, and 109 circulating recombinant forms or CRFs). Using a bioinformatics tool developed in our laboratory (EpiMolBio program), we analyzed the amino acid conservation compared to the HXB2 subtype B reference sequence and the V-markers, or amino acid changes that were specific for each variant with at least 10 available sequences. We inferred the p24 consensus sequence for HIV-1 and for each group to analyze the overall conservation in p24 main structural regions, reporting the percentage of substitutions per variant affecting the capsid assembly and molecule-binding, including those associated with resistance to the new capsid-inhibitor lenacapavir, and the key residues involved in lenacapavir-p24 interaction, according to the bibliography. Although the overall structure of p24 was highly conserved, the conservation in the secondary structure varied between HIV-1 variants and the type of secondary structure. All HIV-1 variants presented >80% amino acid conservation vs. HXB2 reference sequence, except for group M sub-subtype F1 (69.27%). Mutants affecting the capsid assembly or lenacapavir capsid-binding were found in <1% of the p24 consensus sequence. Our study reports the HIV-1 variants carrying 14 unique single V-markers in 9/38 group M variants and the level of p24 conservation in each secondary structure region among the 4 HIV-1 groups and group M variants, revealing no natural resistance to lenacapavir in any HIV-1 variant. We present a thorough analysis of p24 variability among all HIV-1 variants circulating to date. Since p24 genetic variability can impact the viral replication cycle and the efficacy of new p24-based diagnostic, therapeutic, and vaccine strategies, conservation studies must consider all HIV-1 variants circulating worldwide.

## Introduction

The HIV-1 capsid houses the replicative enzymes and viral genomic RNA, protecting them from antiviral factors and cellular sensors of innate immunity (Le Sage et al., 2014), allowing their traffic from entry to near integration sites before fully uncoating (Burdick et al., 2020). Although previous models pointed to an early cytoplasmatic capsid disassembly (Miller et al., 1997; Mamede et al., 2017), recent studies have supported the possibility of a complete or almost intact capsid entering the nucleus (Burdick et al., 2020; Dharan et al., 2020; Li et al., 2021; Zila et al., 2021a). Increasing evidence suggests that the capsid participates in the translocation of viral genomic material into the host nucleus for integration through partial uncoating that allows higher plasticity of the capsid, through structural rearrangements of the nuclear pore, or by both mechanisms (Dharan et al., 2020; Toccafondi et al., 2021; Zhuang and Torbett, 2021; Zila et al., 2021b).

In addition to this structural function, the capsid is involved in different steps of the viral infectious cycle, such as HIV reverse transcription (RT), cytoplasmic trafficking through microtubules, decapsidation and nuclear import of the viral pre-integration complex, integration, and assembly (Sundquist and Kräusslich, 2012; Campbell and Hope, 2015; Novikova et al., 2019; Engelman, 2021; Zila et al., 2021b). Recent *in vitro* studies suggest that the capsid plays a key role in HIV RT contributing directly to its efficiency, maintaining sufficient concentrations of RT, and other core components needed for the long process of RT, excluding cytoplasmatic molecules that may degrade the viral nucleic acids, and interacting with certain molecules, such as IP6 (inositol-hexakisphosphate), which enhances RT *in vitro* by stabilizing the viral capsid (Mallery et al., 2018; Christensen et al., 2020; Aiken and Rousso, 2021). RT requires intact rings or capsid lattice sections with certain stability and geometry to produce double-stranded DNA genomes that are extruded from the ruptured capsid walls (Christensen et al., 2020) while promoting HIV-1 uncoating (Aiken and Rousso, 2021). Moreover, the capsid interacts with multiple host factors that can either promote or prevent virus infection (Stremlau et al., 2006; Fricke et al., 2014; Yamashita and Engelman, 2017; Novikova et al., 2019; Temple et al., 2020; Toccafondi et al., 2021; Wilbourne and Zhang, 2021).

The capsid monomers (p24) are encoded by the *gag* gene segment located between nucleotides 1186 and 1879 of the HIV-1 subtype B HXB2 isolate. The *gag* gene encodes different proteins involved in viral structure and trafficking, assembly, pol protein control, interaction with cellular proteins, and viral egress. Thus, *gag* determines the structure and enzymatic functions in HIV. The main Gag structural proteins are p17 or Matrix, p24 or Capsid (CA) (referred to as p26 in HIV-2), and p7 or Nucleocapsid. These proteins, p6 Gag, and two spacers (p1 and p2) are synthesized from a series of protease-mediated proteolytic reactions at specific cleavage sites (CS) located on the Gag precursor (Pr55*^Gag^*) and GagPol (Pr160^*GagPol*^) polyproteins. An additional ribosomal reading frame during translation of the Gag precursor generates the latter polyprotein (Jacks et al., 1988). Previous reports described different CS conservation across HIV-1 variants (Torrecilla et al., 2014).

The mature capsid structure has the shape of a fullerene cone that consists of approximately 1500 p24 monomers assembled in a hexamer lattice with 12 pentameric variations (Zhao et al., 2013). Each p24 comprises 231 amino acids (aa), has an N-terminal domain (NTD) of 145 aa with a β-hairpin and 7 α-helices (H), a C-terminal domain (CTD) of 85 aa with 4 α-helices, and an 11-residue unstructured region (Zhao et al., 2013). The domains are linked by a flexible linker or interdomain linker region (IDR) (aa 146–150) (Jiang et al., 2011). The NTD is responsible for intra-hexamer contacts, and the CTD forms binding dimers to adjacent hexamers (Rihn et al., 2013). In the center of each hexamer is a pore surrounded by six positively charged arginine residues, and the pore is covered by the β-hairpin that can change conformation to open or close it (Jacques et al., 2016). An IP6 molecule binds to the center of the pore, stabilizing the hexamer (Dick et al., 2018; Mallery et al., 2018; Márquez et al., 2018; Renner et al., 2021). The CA also has a major homology region or MHR (aa 153–172) in the CTD with 20 highly conserved aa (Mammano et al., 1994) and a loop (aa 85–93) in the NTD that binds to cyclophilin A or Cyp A (Gamble et al., 1996).

Previous studies of p24 with mutants generated by targeted mutagenesis have revealed that the capsid is extremely intolerant to non-synonymous substitutions, producing defective or reduced infectivity viruses (Rihn et al., 2013; Perilla and Gronenborn, 2016). This high conservation and the fact that it is the most abundant viral protein make of p24, a very interesting region for the design of serological and molecular diagnostic tests for HIV early detection (Gray et al., 2018; Parekh et al., 2019), is the target of new biosensors and nanotechnologies under development (Kosaka et al., 2017; Zhou and Rossi, 2017; Bala et al., 2018; Farzin et al., 2020; Rodríguez-Galet et al., 2022). In fact, p24 is recognized as an alternative early virological biomarker of infection (Gray et al., 2018). The molecular or serological detection of HIV-1 p24 in diagnostic tests relies on primers, labels, or antibodies binding to conserved areas of the protein across HIV variants, ideally detecting all circulating viral variants (Bbosa et al., 2019). However, naturally occurring aa variations and single aa changes in conserved epitopes can lead to the failure of p24 detection and false-negative results (Beelaert and Fransen, 2010; Ly et al., 2012; Vetter et al., 2015; Qiu et al., 2017). Thus, the evaluation of p24 natural variability, as well as of the performance of each different diagnostic assay across HIV-1 variants, is necessary to identify variants not correctly detected due to viral genetic variability (Alvarez et al., 2015, 2017; Kravitz Del Solar et al., 2018; Stone et al., 2018).

Since the capsid’s stability and integrity are critical to the normal viral replication cycle and infectivity, the HIV capsid is an exciting target for the design of new ARVs (Rihn et al., 2013; Chen, 2016; Jacques et al., 2016; Novikova et al., 2019; McFadden et al., 2021; Saito and Yamashita, 2021). Furthermore, p24 can induce cellular immune responses [Los Alamos National Laboratory (LANL), 2021a] and has been included in some vaccine strategies (Larijani et al., 2018, 2021a,b). Among the numerous molecules that targeted the capsid, lenacapavir seems the most promising to date (Yant et al., 2019; Dvory-Sobol et al., 2022; Margot et al., 2022). Capsid-targeting molecules bind to different sites in p24, aiming to alter the capsid stability and morphology, interfering in the assembly or disassembly processes, or competing with host factors resulting in the suppression of viral infectivity (Li et al., 2013; McFadden et al., 2021).

The HIV is one of the most genetically diverse pathogens due to its high mutation and recombination rates, large population size, and rapid replication rate (Hemelaar, 2012; Hemelaar et al., 2019). The HIV-1 is responsible for most HIV infections worldwide and has been divided into four groups, according to genetic homology: M (major or main), N (non-M, non-O) (Simon et al., 1998), O (outlier) (De Leys et al., 1990), and P (Plantier et al., 2009). Group M is the main HIV group related to the present HIV global pandemic (Hemelaar et al., 2019). This group has been subdivided into 10 subtypes (A-D, F-H, J-L) and 8 sub-sub types (A1, A2, A3, A4, A5, A6, F1, F2) (Robertson et al., 2000; Salminen et al., 2000; Yamaguchi et al., 2020), at least 118 circulating recombinant forms (CRFs) (Los Alamos National Laboratory, 2021b), and countless unique recombinant forms (URFs). The CRF is inter-subtype recombinant viruses detected in three or more not epidemiologically linked individuals (HIV sequence database, 2017), and URF is complex inter-subtype recombinant genomes found only in one HIV infected subject. The emergence of new HIV variants (mainly CRF and URF) and the spread of HIV-1 non-B subtypes and recombinants in this pandemic pose a challenge for the accuracy and efficiency of diagnostic and treatment tools.

Since sequence variation in p24 can influence the phenotypic properties of HIV-1 and its interactions with host factors and ARVs, a deep knowledge of p24 variability across circulating variants and the identification of highly conserved HIV epitopes could help for a more rational design of p24-based diagnostic tests, ARV, and vaccines, which, ideally, should be directed to highly conserved p24 sites across all HIV-1 variants (groups, subtypes, sub-sub types, and recombinants) (Ferrari et al., 2000; Hatziioannou et al., 2004; Thenin-Houssier and Valente, 2016; Saito and Yamashita, 2021). This descriptive study on p24 diversity across secondary structure elements in a large p24 sequence set from different HIV-1 variants reports differences and variant-specific markers in p24 across HIV-1 groups (M, O, N, and P) and group M variants. We also report the proportion of substitutions per variant in p24 residues affecting the capsid assembly (Lingappa et al., 2014), HIV uncoating (Hulme et al., 2015), infectivity (Yamashita et al., 2007; Koh et al., 2013), molecule binding (Qi et al., 2008; Ylinen et al., 2009; McFadden et al., 2021; Saito and Yamashita, 2021), and the new CA-inhibitor lenacapavir key capsid-interaction sites and described resistance mutations (Bester et al., 2020; Link et al., 2020; Sun et al., 2021), according to the bibliography.

## Materials and Methods

In May 2021, we downloaded all available p24 nucleotide sequences from the Los Alamos National Laboratory (2021c), selecting one sequence per patient. The sequences were organized according to their HIV-1 variant in groups, subtypes, sub-subtypes, and CRF. The URF sequences were not included in this study. They were aligned, edited, and translated into aa with the MEGA v6.0 program (Molecular Evolutionary Genetics Analysis^1^) (Tamura et al., 2013) using HIV-1 reference sequence HXB2 (NCBI accession number: K03455.1).

Sequence analysis was performed with an in-house bioinformatics tool (EpiMolBio), previously designed and used in our laboratory for HIV genetic variability analysis and recently updated for severe acute respiratory syndrome-Corona virus-2 (SARS-CoV-2) sequences study (Burgos et al., 2019; Troyano-Hernáez et al., 2020, 2021a,b). This tool is programmed in JAVA OpenJDK version 11.0.9.1 using IDE NetBeans version 12.2. Among other functions, this tool calculates the conservation of a sequence set compared to a reference sequence, as well as the percentage of aa changes for each position within the studied protein. Furthermore, it can infer a consensus from a group of sequences or previously calculated consensus considering the total number of sequences and the frequency of any specific aa residue per position, avoiding the overestimation of polymorphisms present in variants with a small number of available sequences.

We inferred the p24 aa consensus sequence for HIV-1, each HIV-1 group (M, N, O, and P), and each HIV-1 variant (subtype, sub-subtype, and CRF) using all downloaded sequences. Group M consensus was inferred from the consensus of group M subtypes, sub-subtypes, and CRF. The HIV-1 consensus was inferred considering the 4 groups (M, N, O, and P) consensus. The inferred p24 consensus sequences for HIV-1 and each HIV-1 group were used to analyze the mean percentage or level of conserved aa in each p24 residue and each region of its secondary structure. Since the MHR is highly conserved among retroviruses (Gamble et al., 1997), we inferred the HIV-2 p26 consensus sequences by using 182 p26 LANL sequences to analyze this region.

Two analyses were performed only in those variants with at least 10 available p24 sequences to avoid biases due to a low number of sequences. In the first place, we studied the p24 average aa conservation compared to the HXB2 HIV-1 reference sequence in these variants. Secondly, we identified the presence of single V-markers, defined as the natural aa changes specific for each variant and present in >75% of the sequence set for a given position.

We calculated the Wu–Kabat protein variability coefficient (WK) for group M using all available p24 sequences belonging to this group and analyzed the results in the context of the proteins’ domains. The WK coefficient allows studying the susceptibility of an aa position to evolutionary replacements (Kabat et al., 1977). It is calculated using the following formula: Variability = *N* × *k*/*n*, where *N* is the number of sequences in the alignment, *k* is the number of different amino acids at a given position, and *n* is the absolute frequency of the most common amino acid at that position. Therefore, a WK of 1 indicates the same aa was found for that position in all the sequence sets, whereas a WK of >1 indicates relative variability of the respective site, with greater diversity as the WK value increases.

Finally, we identified the substitutions affecting the capsid assembly (Lingappa et al., 2014), HIV uncoating (Hulme et al., 2015), infectivity (Yamashita et al., 2007; Koh et al., 2013), molecule binding (Qi et al., 2008; Ylinen et al., 2009; McFadden et al., 2021; Saito and Yamashita, 2021), and lenacapavir key capsid-interaction sites and described resistance-associated mutations (Bester et al., 2020; Link et al., 2020; Sun et al., 2021) according to the bibliography.

## Results

### p24 Sequences Analyzed and Inferred Consensus Sequences

After discarding incomplete sequences, 23,671 HIV-1 p24 LANL sequences were included in this study (Table 1). Among the HIV-1 p24 sequences, 119 belonged to non-M groups (N, O, and P) and 23,552 were ascribed to group M, including 9 subtypes, 7 sub-sub types, and 109 CRF. The p24 sequences from 9 HIV-1 variants were not available in LANL: CRF30_0206, CRF91_cpx, CRF94_cpx, CRF97_01B, CRF110_BC, CRF111_01C, CRF116_0108, CRF117_0107, and CRF118_BC. We inferred the consensus for each group M variant using all the available p24 LANL sequences for each variant (Table 1). Group M consensus was inferred using the 125 variants consensus. Consensus sequences for groups N, O, and P were generated with the 11, 104, and 4 available p24 LANL sequences for these groups. The HIV-1 p24 consensus sequence was generated using the four HIV-1 groups (M, N, O, and P) consensus. The consensus sequences for group M variants, HIV-1 groups, and HIV-1 inferred with EpiMolBio were aligned (Supplementary Table 1), indicating the most prevalent aa and its percentage of conservation for each p24 residue with a color code.

**TABLE 1:** HIV-1 p24 Los-Alamos National Library (LANL) sequences analyzed in the present study.

HIV-1 variants			N° sequences	HIV-1 variants			N° sequences
		N	11	Group M	CRF	47_BF	5
Non-M groups		O	104			48_01B	6
		P	4			49_cpx	9
Group M	Subtypes	A	61			50_A1D	5
		A1	1408			51_01B	7
		A2	71			52_01B	3
		A3	17			53_01B	4
		A4	3			54_01B	3
		A6	191			55_01B	22
		B	9908			56_cpx	4
		C	4823			57_BC	13
		D	597			58_01B	6
		F	30			59_01B	9
		F1	234			60_BC	8
		F2	31			61_BC	4
		G	176			62_BC	3
		H	15			63_02A	13
		J	7			64_BC	9
		K	4			65_cpx	17
	CRF	01_AE	3782			66_BF1	3
		02_AG	539			67_01B	4
		03_AB	7			68_01B	3
		04_cpx	12			69_01B	7
		05_DF	7			70_BF1	5
		06_cpx	42			71_BF1	16
		07_BC	621			72_BF1	6
		08_BC	296			73_BG	2
		09_cpx	11			74_01B	6
		10_CD	3			75_BF	3
		11_cpx	29			76_01B	2
		12_BF	25			77_cpx	4
		13_cpx	12			78_cpx	3
		14_BG	15			79_0107	4
		15_01B	8			80_0107	3
		16_A2D	4			81_cpx	2
		17_BF	7			82_cpx	6
		18_cpx	8			83_cpx	11
		19_cpx	5			84_A1D	3
		20_BG	4			85_BC	12
		21_A2D	3			86_BC	3
		22_01A1	22			87_cpx	4
		23_BG	2			88_BC	3
		24_BG	11			89_BF	3
		25_cpx	5			90_BF1	11
		26_A5U	4			92_C2U	5
		27_cpx	5			93_cpx	3
		28_BF	5			95_02B	5
		29_BF	8			96_cpx	4
		31_BC	3			98_06B	2
		32_06A6	5			99_BF	3
		33_01B	18			100_01C	3
		34_01B	3			101_01B	3
		35_AD	23			102_0107	2
		36_cpx	4			103_01B	4
		37_cpx	5			104_0107	3
		38_BF	5			105_0108	5
		39_BF	3			106_cpx	6
		40_BF	4			107_01B	6
		41_CD	3			108_BC	5
		42_BF	17			109_0107	2
		43_02G	7			112_0107	3
		44_BF	3			113_01B	4
		45_cpx	10			114_01B	3
		46_BF	8			115_01C	3

*In red, variants with <10 sequences. N°, number; CRFs, circulating recombinant forms (Los Alamos National Laboratory, 2021c).*

### p24 aa Conservation in Its Secondary Structure and Main Regions

**FIGURE 1:**
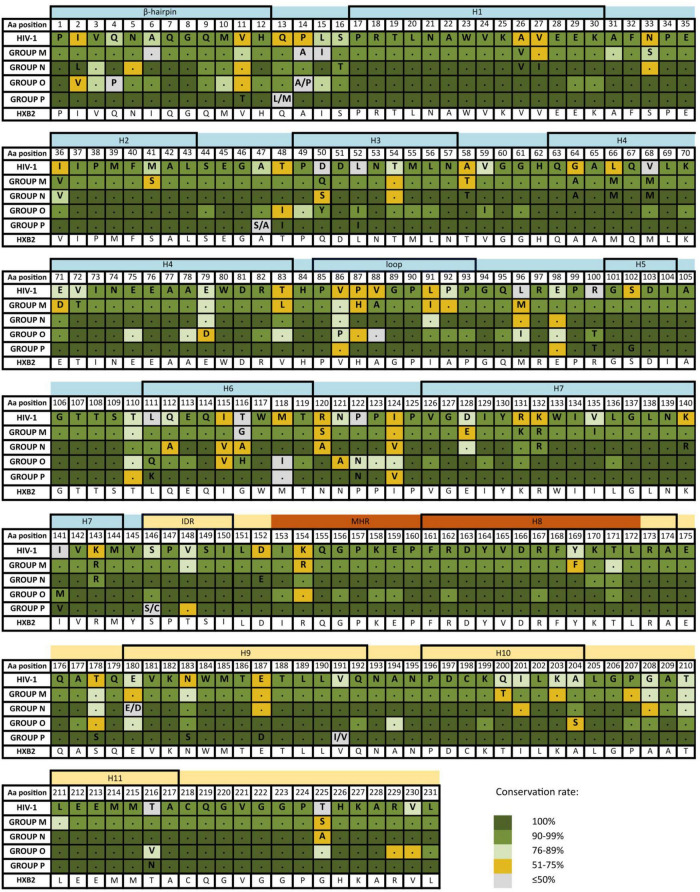
Amino acid conservation percentage along p24 secondary structure in HIV-1 and each HIV-1 group. Aa, amino acid. The aa for each position in groups M, N, O, and P are in reference to HIV-1 consensus. Dots represent the same aa as HIV-1 consensus for that position. The HXB2 reference sequence is described for further guidance below the groups. Colors represent the conservation percentage. P24 domains and secondary structure regions: H, helix; IDR, interdomain linker region; MHR, major homology region; in light blue, N-terminal domain; in light yellow, C-terminal domain; in dark orange, MHR. Aa code: A, alanine; C, cysteine; D, aspartic acid; E, glutamic acid; F, phenylalanine; G, glycine; H, histidine; I, isoleucine; K, lysine; L, leucine; M, methionine; N, asparagine; P, proline; Q, glutamine; R, arginine; S, serine; T, threonine; V, valine; W, tryptophan; Y, tyrosine. Residues P1 and H12 in the β-hairpin and R18 and D51 in the α-helixes 1 (H1) and 3 (H3) are involved in the hexameric pore function.

The inferred p24 consensus sequences for HIV-1 and each HIV-1 group were used to analyze the aa variability of each residue in the p24 secondary structure domains, as illustrated in Figure 1. The HXB2 reference sequence was included for further guidance. The mean p24 aa conservation for the HIV-1 consensus was 89%. For groups M, N, O, and P, it was 94, 96, 95, and 98%, respectively. The mean aa conservation percentage of p24 compared to HXB2 reference sequence across HIV-1 groups was 93.6% (group M), 80.2% (group P), 79.1% (group O), and 85.4% (group N), respectively.

**FIGURE 2:**
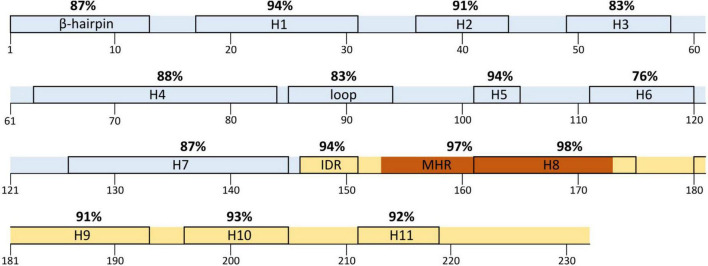
HIV-1 consensus mean conservation of each element in p24 secondary structure. H, helix; IDR, interdomain linker region; MHR, major homology region; in light blue, N-terminal domain; in light yellow, C-terminal domain. The boxes indicate the residues included in each structure except for the MHR, indicated in dark orange.

The aa conservation in the secondary p24 structure varied between HIV-1 variants, amino acid residues, and type of secondary structure. None of the structural regions presented 100% conservation across variants. In HIV-1 consensus, the most conserved structure was helix 8 (98%), followed by the MHR (97%). Both regions overlap at the beginning of p24 CTD (Figure 2). The least conserved region was helix 6 (76%), followed by the Cyp A-binding loop (83%). As for the HIV-1 groups (Tables 2, 3), group P showed the lowest conservation vs. other groups in the IDR (83.3 vs. 94–100%), while group O showed the lowest conservation of the β-hairpin (84.4 vs. 90–100%). Both groups M and O presented lower conservation of the Cyp A-binding loop than groups N and P (86.3 and 86.4 vs. 95.8 and 97.2%). Overall, the Cyp A-binding loop and the β-hairpin were less conserved regions (Figure 1).

**TABLE 2:** Conservation of each element of p24 secondary structure in HIV-1, the four HIV-1 groups, and group M subtype B consensus.

p24 structure	aa sites	HIV-1	Group M	Group N	Group O	Group P	Group M subtype B
β-Hairpin	1–12	86.7	93.0	90.9	84.4	100.0	96.9
H1	17–30	93.8	96.5	100.0	99.8	100.0	97.9
H2	36–43	91.2	96.4	97.7	99.8	100.0	96.4
H3	49–57	83.2	95.2	91.9	95.7	100.0	99.3
H4	63–83	87.8	95.0	98.3	95.4	100.0	98.5
Cyp A loop	85–93	82.8	86.3	95.8	86.4	97.2	90.8
H5	101–104	93.6	99.7	100.0	100.0	100.0	99.8
H6	111–119	75.9	91.9	89.6	86.2	94.4	95.1
H7	126–144	87.1	96.8	97.6	99.3	100.0	98.1
IDR	146–150	94.3	94.1	100.0	99.8	83.3	91.6
MHR	153–172	97.1	95.5	98.6	97.2	100.0	97.9
H8	161–174	97.9	96.5	98.7	99.4	100.0	99.2
H9	180–192	90.9	91.0	93.4	98.7	96.1	96.5
H10	196–204	92.8	90.2	94.5	99.8	100.0	98.9
H11	211–217	92.3	97.6	100.0	96.8	100.0	99.4

*H, helix; IDR, interdomain linker region; MHR, major homology region; Cyp A loop, Cyp A-binding loop; aa, amino acid.*

**TABLE 3:** Range of conservation of each element of p24 secondary structure in the four HIV-1 groups and group M subtype B consensus.

Conservation (%)	Group M	Group N	Group O	Group P	HXB2 subtype B
100		IDR, H1, H11, H5	H5	β-Hairpin, H4, H3, H11, MHR, H7, H8, H2, H10, H1, H5	
98–99.9	H5	H4, MHR, H8	H9, H7, H8, H2, H10, H1, IDR		H5, H11, H3, H8, H10, H4, H7
96–97.9	H11, H7, H8, H1, H2	H7, H2	H11, MHR	H9, Loop	MHR, H1, β-hairpin, H9, H2
94–95.9	MHR, H3, H4, IDR	H10, Loop	H4, H3	H6	H6
92–93.9	β-Hairpin	H9			
90–91.9	H10, H9, H6	β-Hairpin, H3			IDR, Loop
88–89.9		H6			
86–87.9	Loop		H6, Loop		
84–85.9			β-Hairpin		
82–83.9				IDR	

*H, helix; IDR, interdomain linker region; MHR, major homology region; Loop, Cyp A-binding loop.*

**FIGURE 3:**
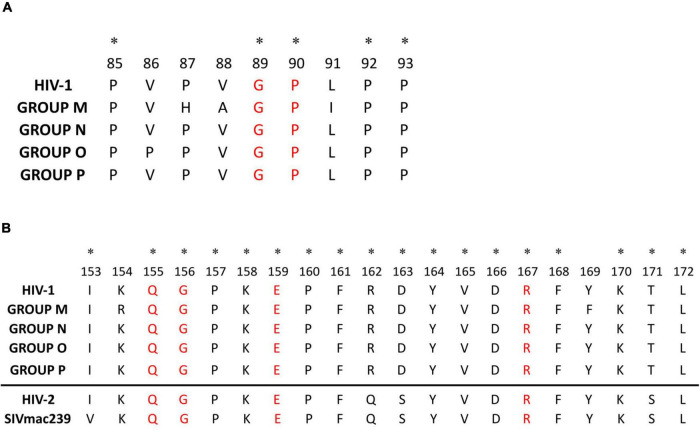
HIV-1 and HIV-1 groups alignment of p24 Cyp A-binding loop **(A)** and major homology region **(B)**. **(A)** Cyp A-binding loop aa alignment (aa 85–93) considering the HIV-1 and the 4 HIV-1 groups consensus sequences. In red, main sites involved in the Cyp A-binding loop interaction; with an asterisk, sites with the same amino acid in all the consensus sequences. **(B)** Mayor homology region aa alignment (aa 153–172) considering the HIV-1 and the 4 HIV-1 groups consensus sequences. The HIV-2 p26 MHR consensus inferred from LANL sequences and SIVmac239 MHR sequence downloaded from LANL have been added below the line for further information. In red, invariable amino acids in retroviruses MHR; with an asterisk, sites with the same amino acid in all the consensus sequences.

The Cyp A-binding loop presented 5 sites sharing the same aa residue in all the consensus: proline in sites 85, 90, 92, and 93, and glycine in site 89 (Figure 3A). The most variable residues were sites 86–88 and 91, showing polymorphisms in group O (V86P) and group M (P87H, V88A, and L91I) compared to HIV-1 consensus alignment. The most variable Cyp A-binding loop sites in group M consensus sequence were 87, 91, and 92 (61.5–69% conservation), with site 91 being the most polymorphic across the 125 group M variants (Supplementary Table 1).

The MHR (sites 153–172) was also highly conserved in the four HIV-1 groups (95–100% conservation) (Figure 3B and Table 2), with 18/20 of its sites showing >90% conservation in the HIV-1 consensus and many sites with complete conservation in groups P (20/20 sites), N (17/20 sites), and O (12/20) (Supplementary Table 1 and Figure 1). The most variable sites in the MHR, with conservation below 75%, were site 154 in groups O and M and site 169 in group M. However, only group M’s aa differed from the general HIV-1 consensus (K154R, and Y169F). Regardless of the level of aa conservation, all the other sites presented the same aa in the consensus sequences (Figure 3B). Sites Q155, G156, E159, and R167, previously described as highly conserved in all retroviruses (Gamble et al., 1997), had ≥99% conservation in all the consensuses.

The main four p24 residues involved in the hexameric pore function (P1 and H12 in the β-hairpin and R18 and D51 in the α-helixes 1 and 3) were highly conserved in HIV-1 consensus (>98%), presenting the same aa in the HIV-1 groups (Figure 1). Groups N and P had complete conservation of these 4 sites. Group O consensus showed 100% conservation in all, except H12 (96% conservation). Group M had conservation above 99% in P1, R18, and D51, whereas H12 had conservation of 97%. In a deeper analysis, the most prevalent mutation in site 12 was tyrosine instead of histidine, found in 0.03% of the group M consensus (683 sequences) and 4 out of 104 group O sequences.

### Capsid aa Conservation Across HIV-1 Variants and V-Markers

**FIGURE 4:**
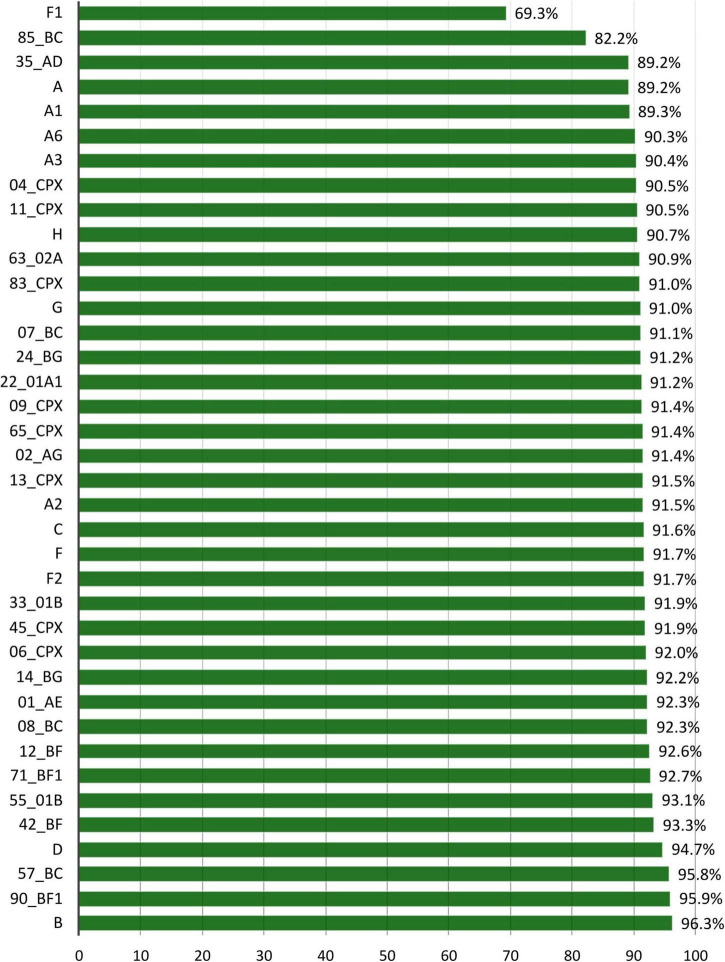
Percentage of aa conservation of p24 across the 38 HIV-1 group M variants with ≥10 p24 sequences at LANL. *X*-axis: HIV-1 group M variants with at least 10 available sequences. *Y*-axis: mean p24 conservation percentage for each variant included in this analysis.

To analyze the p24 conservation across each group M variant and identify the specific V-markers, we used the 38 variants with at least 10 p24 available LANL sequences, corresponding to 7 subtypes (A-H), 6 sub-sub types (A1–A3, A6, F1–F2), and 25 CRF. The F1 was the least conserved subtype (69.3%), and CRF85_BC was the least conserved CRF (82.2%) (Figure 4). All the other subtypes and CRF showed a p24 conservation above 89%.

**FIGURE 5:**
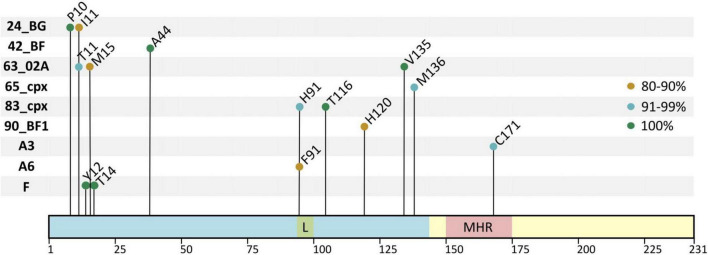
Single V-markers across HIV-1 group M variants. *X*-axis, p24 positions and relevant domains; *Y*-axis, HIV-1 variants carrying single V-markers. In light blue, N-terminal domain, including the Cyp A-binding loop (L) in green. In light yellow, C-terminal domain, including the major homology region (MHR) in red; the conservation percentage of each V-marker is represented in the figure with colored circles (in green, 100%; in blue, 91–99%; in orange, 80–90%). In HXB2 HIV-1 reference sequence the residues present in each site were: M10, V11, H12, A14, I15, S44, I91, G116, N120, I135, L136, and T171. In group M consensus the same residues were present except for S120.

We identified the variant-specific single V-markers present in >75% of the p24 sequences for a given position. Among the 38 HIV-1 group M variants with ≥10 sequences, 9 variants carried 14 single V-markers. Figure 5 shows the 14 V-markers location in p24, the variant they belong to, and their conservation percentage. The legend describes the main residue found in the reference sequence in the corresponding sites.

Subtype F (30 sequences) had two V-markers, Y12 and T14, located in the NTD, both present in all 30 sequences. Sub-subtypes A3 (17 sequences) and A6 (191 sequences) presented one V-marker each: C171 (94.1%) in A3 located in the MHR and the only V-marker in p24 CTD, and F91 (82.7%) in A6 located in the Cyp A-binding loop. Six CRF presented V-markers: CRF63_02A (13 sequences) presented three markers in the NTD: T11 (92.3%), M15 (84.6%), and V135 (100%). The CRF24_BG and CRF83_cpx, both with 11 sequences, presented two V-markers each. In CRF24_BG, we found the V-markers P10 (100%) and I11 (81.8%) located in the NTD β-hairpin. In CRF83_cpx, the V-markers were H91 (90.9%), located in the Cyp A-binding loop, and T116 (100%), located in helix 6, both in p24 NTD. The other three CRF presented one V-marker each. The CRF42_BF (17 sequences) had A44 (100%), CRF65_cpx (17 sequences) had M136 (94.1%), and CRF90_BF1 (11 sequences) had H120 (81.8%) all located in the NTD (Figure 5).

### Wu–Kabat p24 Variability Coefficient in Group M

**FIGURE 6:**
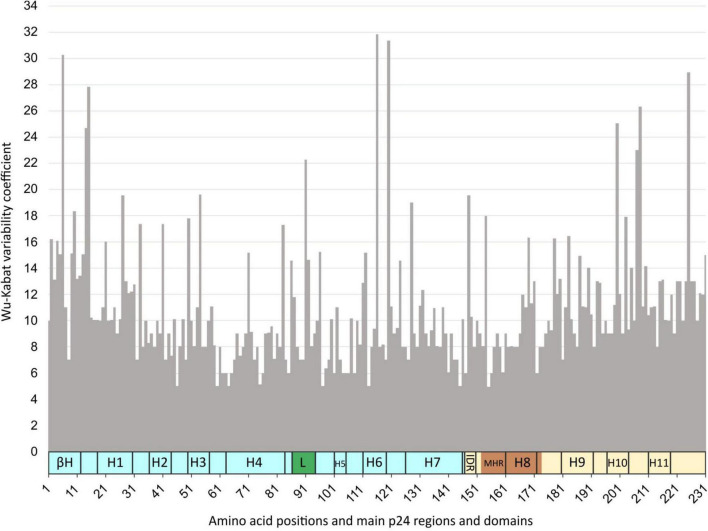
Wu–Kabat (WK) p24 variability coefficient plot in HIV-1 group M sequences. *X*-axis, amino acid positions and main p24 regions and domains; *Y*-axis, Wu–Kabat variability coefficient. βH, β-hairpin; H, α-helix; L, Cyp A-binding loop (green); IDR, interdomain linker region; MHR, major homology region (dark orange); in light blue, N-terminal domain; in light yellow, C-terminal domain.

The median variability coefficient along p24 in group M sequences was 10, being 9 in the NTD and 10.3 in the CTD. The P24 site 116 in H6 presented the maximum coefficient (WK 31.8), followed by site 120 in NTD after H6 (WK 31.4), site 6 in β-hairpin (WK 30), site 225 in CTD end (WK 28.9), and site 15 in NTD before H1 (WK 27.8) (Figure 6). The smallest WK coefficient was 5 found in sites 46 (NTD after H2), 60 (NTD after H3), 64 (H4), 97 (NTD after the Cyp A-binding loop), 113 (H6), 145 (last NTD aa), and 155 (MHR). The median WK in the Cyp A-binding loop was 8 (mean WK 11), with the larger variability in site 91 (WK 22), followed by sites 92, 86, and 87 (WK 14–11). The median variability coefficient of the MHR was the same as in the Cyp A-binding loop, WK 8 (mean WK 9.4), with 22 of its 27 sites (81%) below this value. The most variable sites in MHR were 148 (WK 19.5), followed by 154 (WK 18), and the most conserved was 155 (WK 5). Supplementary Table 2 describes the WK values for each of the 231 p24 residues after the alignment of 23,552 p24 LANL sequences ascribed to HIV-1 group M variants (9 subtypes, 7 sub-sub types, and 109 CRF) under study.

### Capsid Polymorphisms Affecting HIV Phenotypic Properties

Since p24 genetic variability can impact on viral replication cycle at different stages, we analyzed the conservation percentage in these p24 positions affecting the capsid assembly (Lingappa et al., 2014), HIV uncoating (Hulme et al., 2015), infectivity (Yamashita et al., 2007; Koh et al., 2013), and molecule binding (Qi et al., 2008; Ylinen et al., 2009; Saito and Yamashita, 2021).

For the proper assembly of the immature capsid, the functional CA protein must present the residues V181/K182, W184/M185, and L189/L190 in the α-helix 9 (H9) that forms the CTD-end dimer interface between capsid hexamers. The presence of double mutants at these positions alters the hydrophobic bonds that stabilize the interaction of the dimer interface with the homologous partner (Lingappa et al., 2014). In our p24 sequence set, we found no double mutants within H9 in groups N, O, or P. In group M, we found double mutants in some variants (Supplementary Table 3), with an overall percentage below 0.01% for this group.

At the beginning of the CTD, there are 3 aa in non-contiguous regions close to each other in the tertiary structure: K158 (MHR), D197 (H10), and P224, where aa changes can affect Gag multimerization in the membrane. The three residues were well conserved in the O, P, and N groups, and only one group O p24 sequence (0.9%) harbored the aa change K158E. In group M, mutants were found in all 3 positions, again at very low frequency (Supplementary Table 3).

Double mutations at positions E75/E76 in H4, R100/S102 in H5, and T107/T108 (between H5 and H6) and T110/Q112 in H6 inhibit the final step of assembly, leading to the accumulation of pathway intermediates with no virion exit (Lingappa et al., 2014). In group M, double mutants were found in all these positions (Supplementary Table 3), being rare in positions 75–76, which is highly conserved. No changes were found in groups N, O, and P in positions 75/76 and 107/108. Threonine in site 100 was the predominant residue for both groups O and P (Figure 1). Double mutants were only found in group P for these sites, where T100-G102 was present in all group’s P available p24 sequences. Positions 110–112 presented double mutants in all the groups. Alanine is the main aa in site 112 in group N consensus, but only one N sequence presented double mutants in these sites. Site 110 was one of the least conserved sites involving the capsid assembly, and most double mutants had asparagine (N) in this site. However, 110–112 double mutants accounted for less than 1% of the total studied sequences.

Mutants associated with alteration of p24 molecule-binding and HIV-1 infectivity (Saito and Yamashita, 2021) were very infrequent (Supplementary Table 3). One exception was H87Q found in all group M subtypes but J, all sub-sub types but A4 and A5, and in 62 CRF. This aa change was the main polymorphism in 22 variants, being present in 100% of the sequences of CRFs 03_AB, 14_BG, 20_BG, 23_BG, 24_BG, 25_CPX, 27_cpx, 41_CD, 43_02G, 61_BC, 68_01B, 73_BG, 76_01B, 83_cpx, and 84_A1D, in sub-subtype A6, and subtype F. The H87Q was also present in ≥80% of p24 sequences from CRF13_cpx, 32_06A6, 60_BC, 82_cpx, and subtype G. It also appeared in 4% of group O sequences.

The other exception was the change G116A found in all groups but P, being present in 73% of group N sequences and in all group M subtypes except for F, in all sub-subtypes except for A4, and in 50 CRF. The G116A appeared in all (100%) p24 sequences from CRFs 21_A2D, 36_cpx, 60_BC, 62_BC, 64_BC, 65_cpx, 68_01B, 77_cpx, 86_BC, 87_cpx, 92_C2U, 93_cpx, 96_cpx, 103_01B, 105_0108, 106_cpx, 108_BC, 109_0107, and 113_01B. It was also found in ≥80% of the sequences in variants CRF05_DF, 07_BC, 19_CPX, 35_AD, 85_BC, sub-subtype F2, and subtypes C and H.

The key interactions between lenacapavir and the capsid take place in residues N57 (H3), K70 and N74 (H4), and N183 (H9) (Link et al., 2020). The first three sites were highly conserved (>99%) in the HIV-1 and groups M, N, O, and P consensus. However, site 183 showed conservation of 65.5% in the HIV-1 consensus, mainly due to residue variability in groups M and P consensus. Asparagine was the main residue found in N (90%) and O groups (94%), being less conserved in M (77%) and replaced by serine in all (100%) group P sequences. Within the group M variants, glycine was the most prevalent residue in site 183 in subtype F (100%), sub-sub types A1 (60%), F1 (68%), and F2 (69%), and in 18 CRF (Supplementary Table 1). In the other four CRF (36_cpx, 66_BF1, 73_BG, and 89_BF), there was not one consensus aa for this position due to the low number of sequences available in LANL (Table 1), sharing asparagine with glycine, serine, or alanine (Supplementary Table 1). The CRF113_01B had histidine in site 183 in all (100%) of its sequences.

Mutations of Q67H, L56I, M66I, K70N, and N74D/S associated with lenacapavir resistance (Bester et al., 2020; Link et al., 2020; Sun et al., 2021) were rare, found in <0.5% of the sequences in subtypes B, C, D, sub-subtype A1, CRFs 01_AE, 02_AG, and 07_BC (Supplementary Table 3). The exception was a change in N74S, present in 10% of CRF45_cpx sequences with only 10 available sequences in LANL. Double mutants, such as Q67H/N74D and Q67H/T107H, were not found in any of the sequences in this study. None of these lenacapavir-associated resistance mutations were found in groups N, O, or P.

## Discussion

Global geographical patterns in HIV-1 variant distribution are changing due to several factors, including population movements, contributing to an unpredictable HIV-1 pandemic (Peeters and Sharp, 2000). The HIV-1 variants have different global prevalence (Hemelaar et al., 2019) and can present distinct levels of HIV-1 genetic diversity (Abecasis et al., 2009). The HIV-1 group M subtype C is the most prevalent HIV variant in this pandemic, causing around 50% of worldwide infections (Hemelaar et al., 2019). Subtype C is also the most prevalent strain in Southern Africa and India; subtype A in parts of East Africa, Russia, and former Soviet Union countries; subtype B in Europe, Americas, and Oceania; CRF01_AE in Asia; and CRF02_AG in Western Africa (Bbosa et al., 2019). However, HIV genomic sequencing is more extended in economically developed nations, which explains that in our dataset, the most represented HIV-1 variant was subtype B (37.7%), followed by the most abundant variant subtype C (18.4%), and recombinant CRF01_AE (14.4%), according to sequence availability in LANL.

The HIV capsid protein has an essential structural and functional role in the viral replication cycle. Its genetic variability can impact the efficacy of new p24-based diagnostic, therapeutic, and vaccine strategies. Regarding HIV diagnosis, although diagnostic tests were historically developed based on HIV-1 subtype B prototype strains that showed limitations to detect some variants (Loussert-Ajaka et al., 1994), considerable efforts have been made to improve the performance of these assays. Despite this, the performance of certain serological assays is still suboptimal, mainly in countries where many variants are circulating (Aghokeng et al., 2009; Alvarez et al., 2015; Oladokun et al., 2015; Kravitz Del Solar et al., 2018). Thus, it remains vital to gain a deeper knowledge of p24 variability across HIV-1 variants, which could help to explain false-negative diagnostic results in patients with acute and established HIV infection and to develop a more rational design of new p24-based diagnostic tests. Unfortunately, manufacturers do not provide detailed information regarding which part of the viral sequence was targeted in their HIV diagnostic assays, which can differ across assays. However, the provided information in Supplementary Table 1 can help manufacturers and researchers, working in the design of new p24-based molecular and serological diagnostic tests, to identify those HIV-1 variants whose diagnosis could be compromised by viral genetic variability.

Therefore, p24 conservation studies must consider all circulating HIV-1 variants worldwide. We present a thorough analysis of p24 variability among 23,671 HIV-1 p24 LANL sequences belonging to more than 100 different variants, including the three non-M HIV-1 groups and a large number of group M subtypes, sub-sub types, and CRF. The sequences were processed by an in-house bioinformatics tool (EpiMolBio) developed for HIV and SARS-CoV-2 variability analysis. Results were analyzed in the context of the secondary structure of p24, focusing on those residues with the most significant functional or therapeutical relevance. At the same time, we report, for the first time to our knowledge, the single natural polymorphisms in p24 that can be considered as genetic markers of each HIV-1 variant or V-markers. We also provide the consensus p24 sequences for HIV-1, HIV-1 groups, and its variants, revealing differences across p24 residues and structural regions. The consensus sequences of HIV proteins and their conservation studies allow a better understanding of structural, functional, and immunogenic potential differences across HIV-1 groups, subtypes, sub-sub types, and recombinants, and have been previously analyzed in other HIV-1 proteins (Sliepen et al., 2019; Zhang et al., 2021). A previous work by Li et al. (2013) analyzed the degree of *gag* functional conservation in 8 group M subtypes (4 subtypes, 2 sub-sub types, and 2 CRF) across 10,862 sequences. Our study updates and expands the knowledge regarding HIV capsid variability, including 23,671 p24 sequences and all the currently available HIV-1 variants, in the LANL database, including the three non-M groups and 125 group M variants (9 subtypes, 7 sub-sub types, and 109 circulating recombinants forms). Moreover, Supplementary Table 1 summarizes the aa conservation in each capsid residue and each variant to help identify the conservation or consensus aa in any p24 residue and HIV-1 variant of interest in the largest p24 sequence set published to date.

Compared to other HIV proteins, including viral enzymes, p24 is an extremely fragile protein (Rihn et al., 2013), where non-synonymous mutations may drastically reduce its viral fitness. This fragility can be related to the need to maintain its complex structure and its interactions with host proteins. Each p24 monomer must interact with at least three others, while some must adopt slightly different structures to form pentamers that allow the capsid to close (Perilla and Gronenborn, 2016). The p24 structure and assembly mechanisms are complex (Chen, 2016), and the basic geometric principles of the capsid structure are conserved among retroviruses (Aiken and Rousso, 2021). In our study, we observed that the aa conservation in the secondary p24 structure after our sequence analysis varied between HIV-1 variants, amino acid residues, and type of secondary structure. Our results showed high mean aa conservation (>89%) for HIV-1, the four HIV-1 groups, and all group M variants consensus sequences, with only two exceptions: F1 sub-subtype (69.3%) and CRF85_BC (82.2%).

The WK protein variability coefficient was analyzed in HIV-1 group M to study the susceptibility of each aa position to evolutionary replacements. The higher WK values were located around H6 and the β-hairpin in the NTD, except for site 225 at the end of the CTD, although the median variability coefficient for the NTD was lower than for the CTD. The median WK values of the Cyp A-binding loop and the MHR were the same (WK 8), but the MHR sequence had overall fewer variable sites, as expected. A tendency for higher variability was observed at the beginning of the NTD and the end of the CTD, and for less variability between H4 and H8, where the Cyp A-binding loop, the IDR, and the MHR are located.

Previous studies have observed that the β-hairpin, Cyp A-binding loop, and IDR are fairly robust or less conserved regions while the α-helices (mainly H2, H5, H6, and H7) are less tolerant to changes and therefore highly conserved (Rihn et al., 2013). However, when studying the conservation of individual secondary structures, we found that in the HIV-1 consensus, α-helixes 6 and 3 showed the same (83%) or lower (76%) conservation than the Cyp A-binding loop, while the IDR had fairly high conservation of 94%. One of the possible reasons for this discrepancy could be the fact that most studies are centered in group M subtype B, more prevalent in West Europe and the United States (Hemelaar et al., 2019). In the specific analysis of the B subtype consensus, we observed that the IDR conservation dropped to 91.6%, being IDR and the Cyp A-binding loop the least conserved structures, whereas most α-helixes were highly conserved. However, the H6 remained the less conserved α-helix (95%).

The high conservation of p24 also makes it an attractive target for antiretrovirals (Chen, 2016; Novikova et al., 2019; McFadden et al., 2021; Saito and Yamashita, 2021; Dvory-Sobol et al., 2022; Margot et al., 2022). Many capsid-targeting molecules that aim to block HIV-1 infection through different mechanisms of action have been developed (Thenin-Houssier and Valente, 2016; Carnes et al., 2018; Cevik and Orkin, 2019; Bester et al., 2020; Dvory-Sobol et al., 2022). The p24 conservation studies across variants help identify highly conserved regions that may be useful to predict the performance of these new molecules across HIV-1 variants, determine the variability in drug CA-binding sites, and detect if reported drug resistance mutations could be naturally present in certain HIV-1 variants. Some HIV variants, such as HIV-2, present natural polymorphisms related to drug resistance fixed during viral evolution in the absence of antiretroviral therapy, which is maintained over time, providing natural resistance to specific antiretrovirals (Menéndez-Arias and Álvarez, 2014; Troyano-Hernáez et al., 2021a). The provided data in Supplementary Table 1 will allow the analysis of the conservation percentage in specific positions and variants, including p24 residues, that may be associated with resistance to new CA-inhibitors developed in the future.

Among newly developed CA-inhibitors, one of the most promising molecules is lenacapavir (GS-6207), a selective, long-acting subcutaneous or oral p24 inhibitor with a multi-stage activity that inhibits both the early and late stages of the HIV-1 replication cycle (Link et al., 2020; Dvory-Sobol et al., 2022). This first-in-class capsid inhibitor is currently undergoing phase II/III clinical trials (Gilead, 2022). It has shown successful antiviral activity after a single subcutaneous injection (Dvory-Sobol et al., 2022), high potency, and synergy when combined with other ARV, with no cross-resistance with approved drugs (Link et al., 2020). However, phase Ib treatment led to the emergence of Q67H mutation, while other mutations have emerged in *in vitro* selection experiments, with M66I showing the higher resistance (Bester et al., 2020; Link et al., 2020; Sun et al., 2021). In our study, mutations associated with lenacapavir resistance were unfrequent, present in <0.5% of the sequences in 7 HIV-1 variants. Specifically, M66I was found in <0.2% of the sequences in subtypes B and C, and sub-subtype A1 and CRF07_BC, suggesting that natural resistance to lenacapavir, according to the mutations identified to date, is unlikely across HIV variants.

Regarding p24-lenacapavir interactions, the lenacapavir binding site is located in the CA phenylalanine–glycine (FG) binding pocket, a hydrophobic pocket formed by residues from H3 and H4 α-helixes in the NTD and H8 and H9 α-helixes in the CTD of an adjacent subunit in the hexamer (Link et al., 2020; McFadden et al., 2021). Molecules that bind at the FG binding pocket can compete with host factors that also bind in this pocket, negatively impacting nuclear import and viral infectivity (Matreyek and Engelman, 2011; Price et al., 2012). Among the four p24 residues (N57, K70, N74, and N183), with which lenacapavir establishes key interactions (Link et al., 2020), the first three were highly conserved among HIV groups and variants. On the other hand, N183 showed comparatively lower conservation in group M consensus (77%), mainly due to the presence of glycine (non-polar hydrophobic aa) instead of asparagine (polar hydrophilic aa) in some subtypes and variants. The effect of this aa change and its impact on lenacapavir-CA interaction in these variants should be determined with further structural studies.

The HIV-1 p24 interacts with host cellular proteins required for the viral replication cycle, and one of the most relevant interactions is with Cyp A (Braaten et al., 1996a; Campbell and Hope, 2015). The Cyp A is a chaperone with peptidyl isomerase activity that has a general role in the p24 tertiary structure. The CypA-capsid interactions modulate the capsid stability, affect its capacity to bind other cellular factors, and promote HIV-1 replication in human cells (Hatziioannou et al., 2004; Peng et al., 2019; Selyutina et al., 2020). The host protein Cyp A is packaged into the HIV-1 virion through the binding between Cyp A and p24 exposed loop (aa 85–93), mediating a specific interaction with a Glycine in site 89 and a Proline in site 90 (Gamble et al., 1996). Mutations that alter the aa in these sites can affect HIV-1 infectivity (Braaten et al., 1996a), although the role of Cyp A in HIV-1 infectivity varies between groups, being crucial in group M but not always required in group O viruses (Braaten et al., 1996b; Wiegers and Kräusslich, 2002). In our analysis, sites G89 and P90 were highly conserved in all the studied HIV-1 p24 sequences. In all non-M groups’ consensus, site 88, also within the Cyp A active site pocket (Gamble et al., 1996), presented Valine instead of Alanine, the adjacent site 87 presented Proline instead of Histidine, and site 91 had Leucine instead of Isoleucine (Figure 3). In site 86, only group O consensus presented Proline instead of Valine. A previous study reported similar changes in group O isolates, with variable effects across them, suggesting that regions outside the Cyp A-binding loop may also affect the Cyp A effect in HIV-1 replication (Wiegers and Kräusslich, 2002). The most variable Cyp A-binding loop sites in group M were 87, 91, and 92 (60–68% conservation). Again, this reinforces the importance of considering all HIV-1 variants, not just group M subtype B, in such studies.

We confirmed the high level of conservation in all HIV-1 groups of the MHR, a 20 amino acid motif in the CTD region of p24 (aa 153–172), highly conserved in all orthoretroviruses. This could be explained by the fact that it is indispensable for the correct assembly of virions (Gamble et al., 1997), specifically in the stabilization step of the Gag oligomer after association with the membrane, where MHR is part of the intrahexameric interface of the immature capsid (Tanaka et al., 2016). Four MHR sites (155, 156, 159, and 167) are essential for their structural role (Gamble et al., 1997) and remain almost invariable between retroviruses, all presenting >99% conservation in the consensus sequences of HIV-1 and the four groups.

The HIV capsid protects the viral components from cytosolic sensors and nucleases by modulating the capsid hexamers’ positively charged pore, while it allows access to the nucleotides required for efficient RT (Campbell and Hope, 2015; Jacques et al., 2016). Four key aa have been described for the correct functioning of the pore: P1, H12, R18, and D51, where mutations in P1 and D51 have been reported to produce non-infectious viral particles (Jacques et al., 2016). In our study, these sites were highly conserved in all groups and variants, showing relatively lower conservation in H12 in groups M and O, where the most frequent polymorphism H12Y was found in 0.03 and 0.04% of each group’s sequences, respectively. This mutation has been described to favor the closed conformation of the pore, reducing the kinetics of RT (Jacques et al., 2016).

We looked for mutations in p24 related to altered assembly of the immature virus capsid, which were found anecdotally, except for some variants and specific positions, but always in a low proportion (<1% of the total sequences). However, we cannot exclude the possibility that the sequences harboring these mutations belong to non-infectious viruses. Two of the mutations that may affect the interactions between the capsid and capsid-binding molecules (H87Q, G116A) were very frequent in a large number of variants, being considered natural polymorphisms shared between several HIV-1 variants. Q87, located in the Cyp A-binding loop (main consensus aa in most HIV-1 variants), has been associated with reduced Cyp A binding (Yoo et al., 1997; Saito and Yamashita, 2021) and A116 (main consensus residue in 27 HIV-1 variants), with increased infectivity in simian cells (Hatziioannou et al., 2004; Saito and Yamashita, 2021).

We found single specific V-markers in 9 group M variants: CRFs24_BG, 42_BF, 63_02A, 65_cpx, 83_cpx, and 90_BF1, sub-sub types A3 and A6, and subtype F. These markers were conserved in >75% of sequences of the corresponding variant and were unique for that variant. Most of them were found in the capsid NTD. All V-markers found in our study were present at a high proportion of isolates of the corresponding variants, regardless of the sampling year, country, patient, or clinical outcome. Since viral evolution is the result of selective pressures for adaptation over the high diversity of HIV (Bozek and Lengauer, 2010; Peeters et al., 2020), we considered that those markers were fixed during HIV evolution due to different selective pressures when these variants were originated. However, further research is needed to elucidate if these aa changes in p24 residues found in specific variants can affect their p24 function or structure impacting viral fitness, especially those V-markers found in regions with relevant functional implications for the capsid. This would be the case of the V-markers located in the Cyp A-binding loop, H91 (CRF83_cpx) and F91 (sub-subtype A6), and the MHR C171 (sub-subtype A3).

The study’s main limitation was the reduced availability of p24 LANL sequences for some HIV-1 variants. Some of them had less than 10 p24 sequences, which excluded them from the V-marker and variant conservation analysis according to our methods to avoid data overestimation. For instance, the low sequence coverage for group P could explain the absence of the aa change G116A, found in all the other HIV groups. The same could occur in the N183 lenacapavir CA-binding site, where serine replaces asparagine in all group P sequences. The HIV genomic sequencing should be strengthened, especially in developing nations, to enrich the information available on non-B variants through the development of cheaper techniques and international collaboration.

The provided information on p24 variability among all HIV-1 variants circulating to date can be helpful for a more rational design of CA-inhibitors, diagnostic tests, and future HIV-1 vaccines based on the capsid protein. Our results also suggest that natural resistance to lenacapavir (based on the mutations identified to date) is unlikely across HIV variants. Our study also provides the first identification of specific natural polymorphisms of p24 or V-markers that can be considered as specific markers in nine HIV variants. Further studies are required to evaluate the impact of the different levels of aa conservation in p24 across HIV-1 variants and of the specific V-markers found in p24 on the capsid structure, assembly, molecule-binding, and other functions in the viral replication cycle.

## Data Availability Statement

The original contributions presented in the study are included in the article/Supplementary Material, further inquiries can be directed to the corresponding author.

## Author Contributions

PT-H downloaded and analyzed the HIV p24 LANL sequences, validated some EpiMolBio functions necessary for the sequences analyses, performed the computations, discussed the results, and wrote the first version of the manuscript. RR developed the in-house EpiMolBio bioinformatics program and validated the EpiMolBio functions necessary for the sequences analyses. AH designed and supervised the study, discussed the results, reviewed and edited the manuscript, applied for funding, and was responsible for project administration. All authors approved the submitted final version.

## Conflict of Interest

The authors declare that the research was conducted in the absence of any commercial or financial relationships that could be construed as a potential conflict of interest.

## Publisher’s Note

All claims expressed in this article are solely those of the authors and do not necessarily represent those of their affiliated organizations, or those of the publisher, the editors and the reviewers. Any product that may be evaluated in this article, or claim that may be made by its manufacturer, is not guaranteed or endorsed by the publisher.

## Footnotes

1
https://www.megasoftware.net/
